# Renal Transplantation Dramatically Reduces IgA Anti-beta-2-glycoprotein I Antibodies in Patients with Endstage Renal Disease

**DOI:** 10.1155/2014/641962

**Published:** 2014-04-10

**Authors:** Manuel Serrano, Jose Angel Martínez-Flores, Maria José Castro, Florencio García, David Lora, Dolores Pérez, Esther Gonzalez, Estela Paz-Artal, Jose M. Morales, Antonio Serrano

**Affiliations:** ^1^Servicio de Nefrología, Instituto de Investigacion Hospital Universitario 12 de Octubre, Avenida Córdoba s/n, 28041 Madrid, Spain; ^2^Servicio de Inmunología, Instituto de Investigacion Hospital Universitario 12 de Octubre, Avenida Córdoba s/n, 28041 Madrid, Spain; ^3^Servicio de Epidemiología, Instituto de Investigacion Hospital Universitario 12 de Octubre, Avenida Córdoba s/n, 28041 Madrid, Spain; ^4^Sección de Inmunología, Universidad San Pablo-CEU, Campus de Monteprincipe, 28668 Madrid, Spain; ^5^Facultad de Medicina, Universidad Complutense, 28040 Madrid, Spain

## Abstract

IgA anti-beta-2-glycoprotein I (aB2GPI) antibodies have been related to vascular pathology in the general population and mainly in hemodialyzed patients (prevalence 33%) in whom an elevated incidence of thrombosis and mortality is found. In this paper we have studied the presence of IgA aB2GPI antibodies at pretransplant and their evolution after transplantation with a cross-sectional-based follow-up study of a cohort of 288 endstage renal disease (ESRD) patients treated with kidney transplantation. Pretransplant IgA aB2GPI levels were elevated 31.7 ± 4.2 U/mL without differences in age or type of dialysis. Patients with different etiologies of ESRD showed higher levels of IgA aB2GPI than blood donors, except the groups of non-IgA glomerular disease and systemic erythematosus lupus, whose nonsignificant differences were observed. IgA aB2GPI antibodies dropped immediately after transplantation (10.7 ± 1.0 U/mL, *P* < 0.0001), coinciding with a high degree of immunosuppression, and remained significantly lower than that observed in pretransplant status. Prevalence of patients with elevated antibodies was also less in transplanted patients (8.9% versus 30.4%, *P* < 0.0001). Among, positivity for IgA aB2GPI was higher than in patients who had received their first transplant that those were retransplanted. This finding could have important clinical implications and can suggest new therapeutic strategies in patients with IgA aB2GPI antibodies.

## 1. Introduction

Prevalence of cardiovascular disease is higher in chronic kidney disease (CKD) patients than in the general population. This is especially important in dialyzed patients who have frequent cardiovascular complications [1–3], including thrombotic episodes that lead the causes of death in these patients [4].

Patients who have received renal transplantation have shown a significant reduction in cardiovascular morbidity and death [5]. However, there is a higher incidence of cardiovascular disease in transplanted patients than in the general population and it is still the major known cause of death in kidney transplant patients [5, 6].

Antiphospholipid antibodies (aPL) are a heterogeneous group of autoantibodies directed against phospholipids, phospholipids binding proteins, or both together. Antigens recognized by aPL are located on the membranes of cells involved in the coagulation cascade [7]. aPL associated with vascular pathology are directed against protein *β*2-glycoprotein I (B2GPI) [8], a serum apolipoprotein that is also expressed in the membranes of platelets, endothelial cells [9], and kidney tubular epithelium [10, 11].

Antiphospholipid syndrome (APS) is defined by the persistent presence of aPL associated with clinical criteria for the diagnosis of APS. These criteria are the inclusion of one or more episodes of arterial, venous, or small vessel thrombosis in any tissue or organ or pregnancy morbidity. Laboratory criteria are presence of lupus anticoagulant, anti-cardiolipin (aCL), or anti-*β*2-glycoprotein I (aB2GPI) antibodies (IgG or IgM isotypes) in serum or plasma [12].

aB2GPI antibodies of IgA isotype have been related to APS [13]. They have also been described as an independent risk factor for cardiovascular disease, acute myocardial infarction, acute cerebral ischemia, and atherosclerotic disease [14].* In vivo* mouse studies have demonstrated that IgA aB2GPI antibodies are not an epiphenomenon but rather are directly prothrombotic [13]. However, the current consensus criteria for diagnostic antibodies on APS only include IgG and IgM antibodies. aB2GPI antibodies of IgA isotype are not included because there is disagreement on the meaning of this biomarker. The controversy is mainly because diagnostic kits with differences in sensitivity and specificity are used [15, 16].

Presence of aPL (IgG and IgM isotypes) is more frequent in patients with chronic kidney disease from any cause than in the general population [17–19]. It is independent of age, length of time on dialysis, sex, type of dialysis membrane, drug treatment, and hepatitis B or C virus infection [20].

The origin of these antibodies is unknown. However, there has been speculation regarding the role of the dialysis membranes [9], repeated endothelial injury involved in dialysis system access, and microbial infections [21–23]. Nonetheless, the association of consensus aPL with thrombotic events is uncertain as there are studies both for and against it [21, 24, 25]. This has led some authors to question if these antibodies are truly pathogenic or are just an epiphenomenon [26].

Our group recently described an increased prevalence of aB2GPI antibodies of IgA isotype in hemodialyzed patients (33%) and a clear association with thrombotic events and mortality [27]. This finding was subsequently confirmed by other authors [28]. However, the prevalence of CKD in different stages and evolution of these autoantibodies after transplantation have not been clearly defined.

In this paper, we have studied the presence of IgA aB2GPI autoantibodies before transplantation and its evolution after transplantation, including the two possible patterns of evolution: (1) stable renal function and (2) graft loss with return to hemodialysis and retransplant. We have shown that the IgA aB2GPI antibody levels drop immediately after transplantation and that this decline persists over time, even in patients who have lost their graft and returned to hemodialysis.

## 2. Methods

### 2.1. Study Design

This is a cross-sectional-based follow-up study of a cohort of endstage renal disease (ESRD) patients treated with kidney transplantation.

*Main Endpoint*. To investigate the evolution of IgA-aB2GPI antibodies within the endstage renal disease status up to the first years after transplant.

*Secondary Endpoints*. To investigate the association of IgA aB2GPI antibodies with age, cause of chronic kidney disease, improvement of renal function, and return to renal failure after graft loss.

### 2.2. Patients

(1) Posttransplant followup of a group of patients who had received a kidney transplant in the previous four years: a total of 288 transplanted patients (56.2% males) were recruited randomly by physicians at the time of a posttransplant scheduled health screening. The patient enrollment process and sample shipment were performed from January 2011 to March 2012. On enrollment, patients were clinically stable (creatinine less than 2 mg/dL) and following an immunosuppression protocol based on calcineurin inhibitors plus mycophenolate. Ethnicity breakdown was 280 Caucasians, two Asians, and six East Africans. At the moment of transplant, mean age was 52.0 years ± 0.89. Of the 288 patients, 63 were younger than 40 years, 113 were between 40 and 60, and 112 were older than 60. Clinical situation: 235 were undergoing hemodialysis, 32 were receiving peritoneal dialysis, and 21 were undialyzed (predialysis). A total of 258 were first transplants and 30 (10.4%) were retransplants.

All the patients were tested for pretransplant antibodies using the serum sample used in crossmatch.

(2) Control population: A control group made up of 220 anonymous healthy blood donors were used as representative of the general population.

### 2.3. Immunosuppressive Treatment

The immunosuppressive protocol used was based on calcineurin inhibitor tacrolimus, associated with steroids and mycophenolate mofetil (MMF) with or without biologic drug induction [29].

*Induction*. Antithymocyte immunoglobulin: in patients with immunological risk, initial immunosuppression induction was performed with rabbit antithymocyte (1.5 mg/kg for 4 days) and corticosteroids, 500 mg of methylprednisolone before graft reperfusion. On the first posttransplant day, methylprednisolone 125 mg/8 h was administered, followed by 125 mg/12 h on the second day and 125 mg on the third day. The following drugs were administered from the fourth day on: prednisone: 1 mg/kg/day; calcineurin inhibitor: tacrolimus 0.1 mg/kg/day (levels 10–15 ng/mL); MMF 1 g/day. 

*Maintenance*. The corticosteroid doses were progressively reduced in all the patients in the second quarter. The dose was totally discontinued in the case of low-risk patients, with the remaining patients continuing with low doses. Tacrolimus was reduced gradually from the second quarter until reaching levels between 4 to 10 ng/mL. MMF dose was adjusted in the second quarter, until reaching target trough mycophenolic acid levels between 2 and 5 ng/mL.

### 2.4. Antibodies

Autoantibodies IgA IgG and IgM aB2GPI and anti-cardiolipin were quantified by enzyme-linked immunosorbent assays QUANTA Lite (INOVA Diagnostics, San Diego, CA). Autoantibodies were considered positive with values >20 U/mL according to the manufacturer's guidelines.

### 2.5. Statistical Methods

Results are expressed as mean ± standard error (SEM) or absolute and relative frequencies. Comparisons of the distributions of continuous measurements were made using the Wilcoxon-Mann-Whitney test or Student's *t*-test, as appropriate. Differences between pretransplant and posttransplant measures were evaluated with a paired *t*-test.

Results were considered significant with probabilities less than 0.05.

Data were processed and analyzed using the statistical program STATA 11 (StataCorp LP, College Station, TX, USA).

### 2.6. Ethical Issues

The study was approved by the Institutional Review Board of the Hospital 12 de Octubre.

## 3. Results

### 3.1. Patients with Pretransplant IgA aB2GPI Antibodies

Eighty-one patients (28.8%) were positive for IgA aB2GPI antibodies in the pretransplant sample. Mean levels were 22.40 ± 2.58 U/mL.

**Figure 1:**
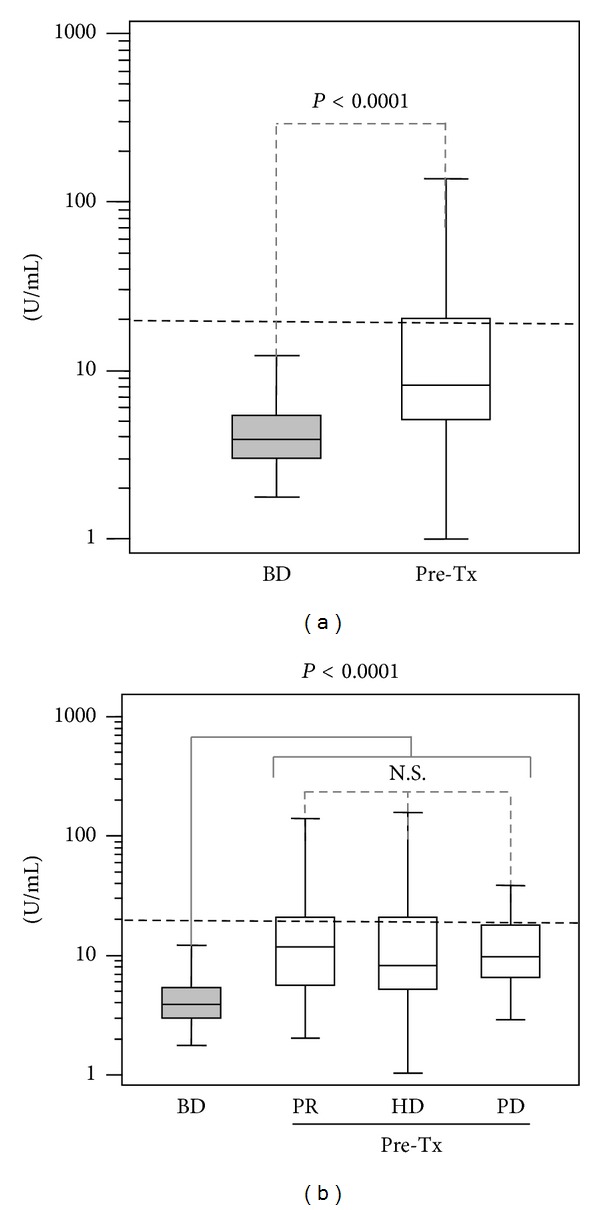
(a) Pretransplant levels of IgA aB2GPI in all the patients versus blood donors. (b) Pretransplant levels of IgA aB2GPI in the three subgroups of treatment of renal failure prior to transplantation versus blood donors. BD: blood donors. Pre-Tx: All samples pretransplant. PR: undialyzed patients (predialysis). HD: patients on hemodialysis. PD: patients on peritoneal dialysis. Cut-off is shown with a dotted horizontal line.

In the control group, 3 blood donors were positive (1.4%). Mean levels were 5.10 ± 0.44 U/mL (mean/SEM), with the differences being clearly significant (*P* < 0.0001, Figure 1(a)).

### 3.2. Clinical Condition Pretransplant and IgA aB2GPI Antibodies

Prevalence of patients positive for IgA aB2GPI antibodies in the subgroups treated with hemodialysis (65/235) peritoneal dialysis (8/32) and undialyzed patients (8/21) were similar (*P* = 0.5454). Levels of IgA aB2GPI antibodies in the three subgroups versus blood donors were clearly significant (*P* < 0.0001). However, these levels were not significant between the three subgroups (Figure 1(b)). Creatinine levels in undialyzed patients were 4.9 ± 0.27 mg/dL (mean/SEM).

As we did not find differences between the three subgroups, we decided to analyze all the patients as a single group.

The etiology of ESRD of all the patients and proportion of patients of each condition positive for IgA aB2GPI antibodies are shown in Table 1. All the groups showed significantly higher levels of IgA aB2GPI and prevalence of positive patients for this antibody than blood donors except the groups of Non-IgA glomerular disease and systemic erythematosus lupus, whose differences were nonsignificant (Table 1).

No significant differences were observed between pretransplant positivity of IgA aB2GPI antibodies and diverse causes of ESRD (not shown) except in patients with non-IgA glomerulopathy in whom the prevalence of these antibodies compared to the other patients was significantly lower (6.3% versus 30.9%, *P* = 0.0028).

**Figure 2:**
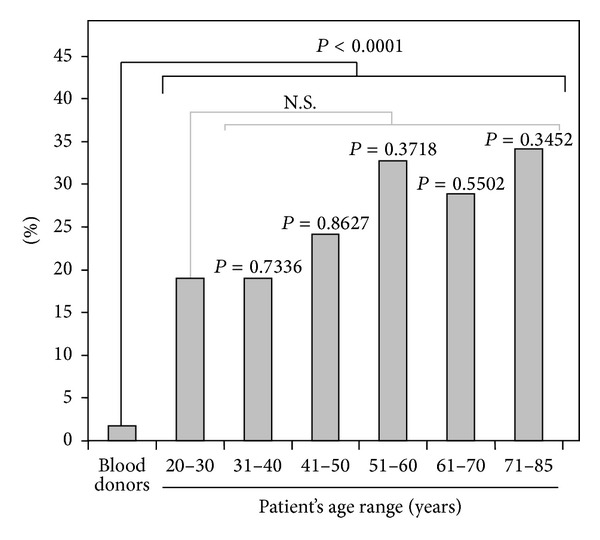
Prevalence of IgA aB2GPI antibodies in age groups of transplanted patients versus blood donors.

Distribution of patients in age groups and prevalence of IgA aB2GPI antibodies in each group are described in Table 2. All age groups had a higher prevalence of IgA aB2GPI antibodies than the blood donors (odds ratio >17, *P* < 0.0001, Table 2, Figure 2).

**Figure 3:**
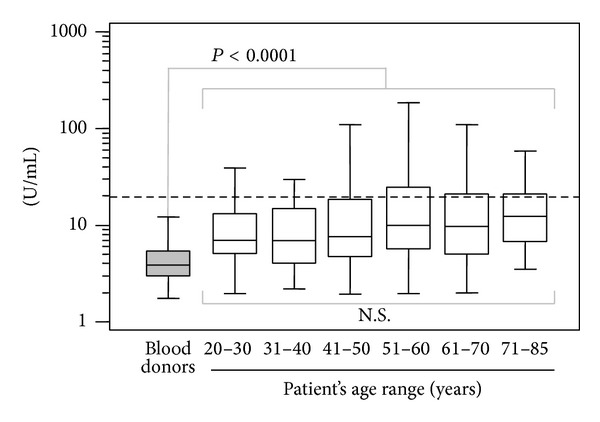
Levels of IgA aB2GPI antibodies by age range in pretransplant samples versus blood donors. Cut-off is shown with a dotted line.

Mean levels of IgA aB2GPI antibodies in all the age groups were significantly elevated compared to the general population in all the age ranges (*P* < 0.0001, Figure 3).

12.5% of patients positive for IgA aB2GPI had a history of major thrombotic events (excluding vascular access) compared to 4.3% of negative (*P* = 0.0282).

### 3.3. Posttransplant Evolution of IgA Antibodies aB2GPI

**Figure 4:**
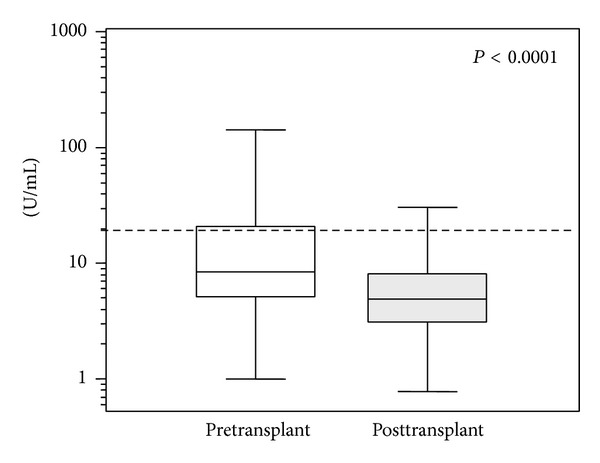
Pretransplant and posttransplant levels of IgA aB2GPI antibodies (*t*-test for paired samples). In patients with more than 1 sample, only the first sample was analyzed. Cut-off is shown with a dotted line.

Posttransplant levels of IgA aB2GPI antibodies were significantly lower (mean 9.4 ± 1.0) than those observed in the same patients in the pretransplant sample (pared samples, *P* < 0.0001) (Figure 4).

**Figure 5:**
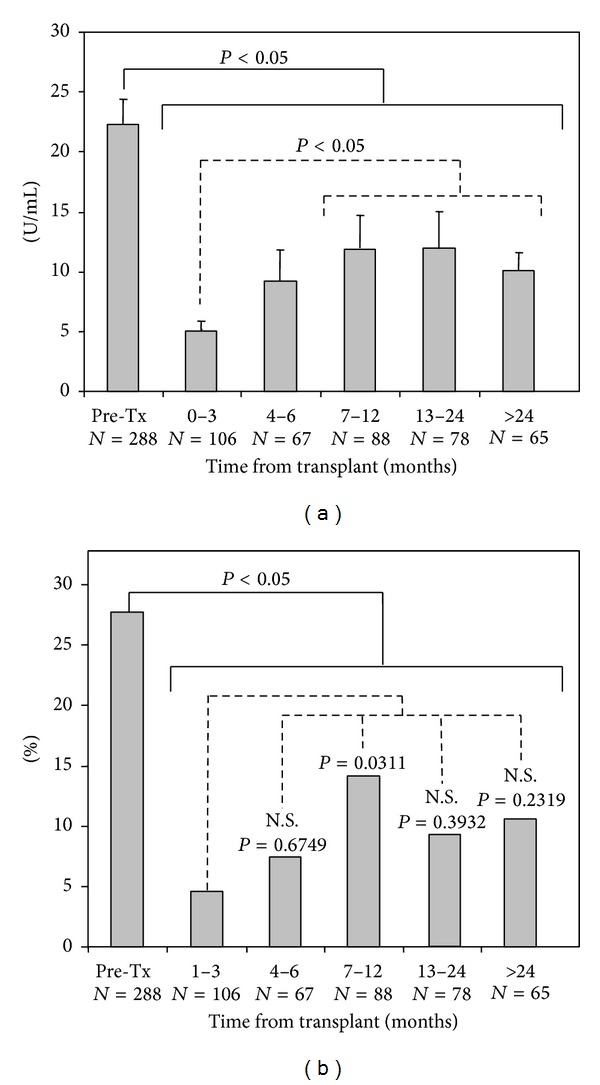
IgA aB2GPI antibodies in posttransplant serum samples grouped in time periods after transplant. (a) Mean levels of antibodies (U/mL) compared with pretransplant samples. The first trimester (period of less levels) compared with the other periods. (b) Percentage of sera positive for IgA aB2GP compared with pretransplant. The first trimester period of lower levels is also compared with the other periods. Tx: transplant.

When serum samples were grouped according to time periods after transplant, mean levels of posttransplant IgA aB2GPI antibodies were as follows: first quarter 5.0 ± 0.6 U/mL (*P* = 0.0006); second quarter: 9.2 ± 2.7 U/mL (*P* = 0.0005); second semester: 11.9 ± 2.8 U/mL (*P* = 0.0065); second year 12.1 ± 3.0 U/mL (*P* = 0.0105); more than two years: 10.4 ± 1.6 (*P* = 0.0001) (Figure 5(a)). The sharp drop in levels in the first quarter was followed by a significant recovery (versus the first quarter) in the second semester beginning with the seventh month posttransplant (*P* < 0.05, Figure 5(a)).

Mean levels of IgA aB2GPI antibodies in blood donors did not differ significantly regarding that observed in the transplanted patients in the first quarter (*P* = 0.9498) and the second quarter posttransplant (*P* = 0.1313).

There were significant differences in the second semester (*P* = 0.0179), second year (*P* = 0.0253), and the group with more than 2 years (*P* = 0.0015).

The percentage of positive patients for IgA aB2GPI also increased significantly (*P* = 0.0311) in the second half and then subsequently dropped and stabilized at approximately 10% (Figure 5(b)).

There were significantly fewer patients positive for IgA aB2GPI antibodies at posttransplant (8.9%) than in pretransplant (28.1%, *P* < 0.0001). When serum samples were grouped in time periods after transplant, positivity was always significantly lower (Figure 5(b), *P* < 0.05) than in the pretransplant. In the first quarter, 4.7% (*P* < 0.0001) were positive; in the second quarter, 7.5% (*P* = 0.0007). In the second semester, 14.8% (*P* = 0.0168) were positive; in the second year, 9.0% (*P* = 0.0008) and after more than two years 10.8% (*P* = 0.0057) were positive. The drop in positivity observed after the first quarter was only significant in the second semester (*P* = 0.0311, Figure 5(b)).

Persistent IgA aB2GPI antibodies after transplant were more frequent in older patients. However posttransplantation levels were significantly lower than those observed before transplantation. No significant differences were observed between causes of ESRD, induction therapy, waiting time, or previous transplants (Table 3).

### 3.4. Other APS Antibodies

**Figure 6:**
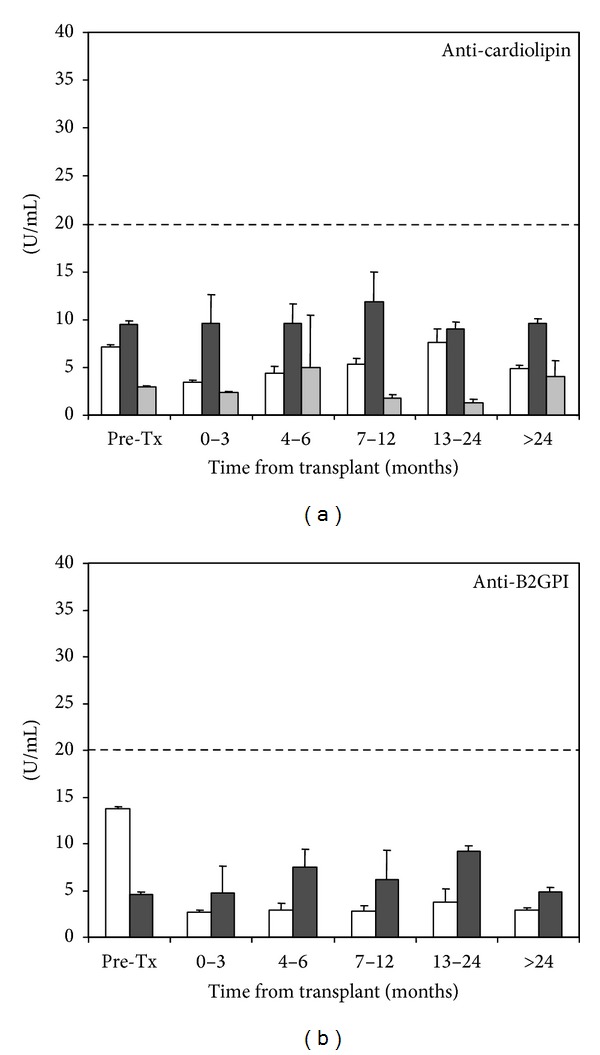
Evolution of aPL antibodies in posttransplant serum samples grouped in time periods. (a) Anti-cardiolipin IgG (white). IgM (dark) and IgA (gray) isotypes. (b) aB2GPI antibodies IgG (White) and IgM (dark). Cut-off is shown with a dotted line.

Posttransplant levels of aCL (IgG, IgM and IgA) and aB2GPI (IgM) did not differ significantly from that observed in pretransplant samples (Figures 6(a) and 6(b)). Although the mean levels of aB2GPI of IgG isotype were not elevated in the pretransplant period, they significantly dropped in posttransplant (Figure 6(b), *P* < 0.0001). No differences were observed in the percentage of positive patients in pre- and posttransplant for these antibodies (not shown).

### 3.5. First Transplant versus Retransplant

Pretransplant levels of IgA aB2GPI antibodies were significantly higher in patients who had received the first transplant that those who received the second/third transplantation (23.8 ± 2.90 versus 10.53 ± 1.84, *P* = 0.0001). Time on the waiting list in patients who received the first renal transplantation was 28.2 ± 5.2 mo. versus 39.1 ± 4.3 mo. in those who were retransplanted (N.S.).

Pretransplant levels of IgA aB2GPI antibodies for retransplanted patients did not show significant difference (*P* = 0.5097) regarding the posttransplant levels in patients who had received their first transplant.

Prevalence of IgA aB2GPI positivity in first transplant patients was higher (29.8%, odds ratio 2.77) than retransplanted patients (13.3%). However, this difference was not significant (*P* = 0.0912).

## 4. Discussion

IgA aB2GPI antibody levels are elevated in CKD patients independently of their clinical condition (peritoneal dialysis, hemodialysis, and undialyzed). The percentage of those positive for IgA aB2GPI antibodies reported in this study is similar to that previously described in hemodialysis patients [27] and in healthy controls [30].

The incidence of thrombotic events before transplantation was 12.3% (excluding fistulae thrombosis) and 4.3% among IgA aB2GPI positive and negative patients, respectively (*P* = 0.028). The incidence in IgA aB2GPI positive patients is lower than what we published before [27] because in the present paper we only examined dialysis patients in the waiting list while in the referenced work all patients on dialysis were evaluated.

IgA aB2GPI antibodies drop immediately after renal transplantation and remain significantly lower than the levels observed in the pretransplant status, even in patients who have lost their graft and have returned to dialysis. We are reporting the evolution of IgA aB2GPI antibody levels in patients with CKD, replacement therapy (peritoneal or hemodialysis), and renal transplantation for the first time.

It is unknown how the immune response of IgA antibodies against B2GPI is generated. Proposal models of hapten-carriers complexes generated by B2GPI interaction with dialysis membranes and endothelial injury by dialysis system access to body [21–23, 27] could be discarded because the prevalence of IgA aB2GPI antibodies in CKD patients in predialysis or those undergoing peritoneal dialysis is similar to the prevalence observed in hemodialysis patients. We could hypothesize that dysfunction of the kidney, an organ that physiologically elaborates B2GPI, may condition the production of atypical B2GPI (misfolding) that could favor the exposure of previous occult epitopes that are similar to microbial epitopes. Molecular mimicry between B2GPI and microbial epitopes, in the context of mucosal infections, may trigger an antibody immune response against B2GPI, with this being biased towards the production of IgA [31].

If the misfolding hypothesis is correct, it could be expected that patients with chronic dysfunction of organs that physiologically produce B2GPI, as the liver and heart, may also have a higher prevalence of aPL, including the IgA isotype. Although there are some reports that could support this hypothesis, epidemiological studies are needed to demonstrate this possibility [32–34].

Isolated IgA aB2GPI antibodies positivity is associated with an increased risk for thrombosis in patients. This statement has been confirmed “*in vivo*” using mouse models [13]. Hemodialysis patients have an elevated incidence of thrombotic events and cardiovascular morbidity [27]. However, when they receive a kidney transplant, thrombotic events are only concentrated within the first posttransplant weeks [35, 36]. In general, the thrombophilic risk factors are corrected within one month after transplantation [37].

Notably, patients with pretransplant IgA aB2GPI antibodies have an elevated risk of early graft loss when they are transplanted, mainly due to thrombosis during the first posttransplantation weeks (Morales et al., unpublished data, submitted).

This lower morbidity after transplantation can be derived from uremia normalization and it would also contribute to the dramatically sharp decline of IgA aB2GPI antibodies.

Antibody levels decline quickly, with this being very pronounced, during the first posttransplantation quarter. This could occur up to the point that the average levels of antibodies in this period become similar to those observed in blood donors. Uremia improvement represents a more physiological situation for the immune system but this, by itself, would not explain the decline in antibodies. This period is associated with a high degree of immunosuppression.

The consequence of immunosuppressive therapy is interference with the immunological memory. This means that the secondary antibody response is depressed in renal allograft recipients [38, 39]. Decreased antibody production coupled with short IgA half-life (5 days) [40] would explain the rapid drop in the antibody level.

Interestingly, this sharp decline was followed by an increase in positivity from the second trimester, a period in which immunosuppressive drugs are gradually reduced to reach maintenance dose from the second semester. It is also of interest that the patients with CKD due to non-IgA glomerulonephritis had similar IgA aB2GPI antibodies as the negative controls. An explanation for this finding could be that these patients received immunosuppressive therapy while patients with other causes of ESRD generally did not receive immunosuppression. Thus, immunosuppressive drugs would block autoantibody production.

It is noteworthy that lower levels of IgA aB2GPI antibodies and a lower percentage of positivity were found in the retransplanted patients than in the first transplants and pre- and posttransplants. The role that immunosuppressive therapy could play in this finding merits future investigations.

The most common treatments for APS treatment are based on anticoagulation in order to prevent thrombotic events. The most accepted protocols for the treatment of APS have only included patients with a history of thrombotic events and have been performed with life-time anticoagulation therapy with a vitamin K inhibitor in order to maintain the international normalized ratio (INR) between 2.0 and 3.0 [41]. These anticoagulant protocols have several variants; among them is the addition of antiplatelet aggregants to patients with a history of arterial thrombosis [42], use of hydroxychloroquine [43], and replacement of vitamin K antagonists in pregnant women by unfractionated heparin plus low-dose aspirin [44]. Preventive anticoagulation treatment is controversial since it conditions any therapeutic decision, such as modifying the dosage or combining it with other drugs. Changing the antithrombotic coverage (with the possibility of it becoming insufficient or excessive) supposes a risk, with the consequent adverse effects [45, 46].

Studies about asymptomatic patients with antibodies aPL (isotypes IgG-IgM) have shown an annual thrombosis risk ranging from 0% to 3.8% [47]. Thromboprophylaxis is only recommended in these subjects in high-risk situations [48]. However, these studies are limited. They do not consider the isotype IgA and have predominantly included patients with systemic lupus erythematosus [49].

Treatment of other antibody-mediated autoimmune disorders, as myasthenia or pemphigus, includes corticosteroids, immunosuppressants (azathioprine, calcineurin inhibitors, or mycophenolate mofetil), and biological therapies [50–52].

Although there are references in the literature on the use of biological immunosuppression in APS, conventional immunosuppression experience has been limited to secondary APS in the context of autoimmune disease treatment [53, 54].

The purpose of the current treatment of APS is not that of eliminating these antibodies, even though they are considered to be the cause of the illness. For this reason, several authors have proposed protocols using rituximab to eliminate these autoantibodies [55]. Good results have been described for both APS and Sneddon syndrome (catastrophic APS) [56]. However, this therapeutic approach is rarely used in the clinical practice and almost only in those patients in whom conventional therapy has been ineffective [57].

This work has several limitations. One limitation is that this study aimed to know the evolution of autoantibodies after renal transplantation in a selection of patients who maintained stable renal function in different periods after transplantation. This represents a bias because we made a positive selection of patients. Consequently, another limitation is that we cannot establish true conclusions concerning the presence of autoantibodies and their relation with the clinical course because patients with important morbidity or those who had lost their kidneys were excluded. These aspects should be addressed in a longitudinal study with a long followup.

In summary, in spite of these limitations, our study has clearly demonstrated that the levels if IgA aB2GPI antibody titers dramatically decrease in the first quarter after renal transplantation, coinciding with improvement of renal function and the high degree of immunosuppression. After that, these antibodies slowly increase, although they do not reach statistical significance and they continue to be lower than pretransplant levels thereafter. If confirmed, these findings could suggest new therapeutic strategies in patients with APS. For these reasons, controlled and randomized studies would be mandatory in patients positive for IgA aB2GPI antibodies in whom there is no therapeutical approach.

## Figures and Tables

**Table 1:** Etiology of endstage renal disease.

Disease	Patients (*N*/%)	IgA aB2GPI positive*	*P* value	IgA aB2GPI (U/mL)**	*P* value
**Blood donors**	**220**	**3 (1.4%)**		5.1 ± 0.44	
Diabetic nephropathy	68 (23.6%)	24 (35.2%)	<0.0001	33.6 ± 8.04	<0.0001
Glomerular disease	50 (16.0%)	8 (16.0%)	<0.0001	9.6 ± 1.39	0.0001
Non-IgA glomerulonephritis	31 (10.7%)	2 (6.4%)	0.1165	6.6 ± 0.85	0.1951
Berger's disease (IgA nephritis)	19 (6.5%)	6 (31.5%)	<0.0001	14.1 ± 3.06	<0.0001
Nephroangiosclerosis	26 (9%)	10 (38.4%)	<0.0001	26.3 ± 6.52	<0.0001
Systemic lupus erythematosus	5 (1.7%)	1 (20%)	0.0865	8.2 ± 4.24	0.2926
Interstitial nephritis	14 (4.8%)	3 (21.4%)	0.0003	33 ± 20.29	<0.0001
Polycystic kidney disease	31 (10.7%)	6 (19.3%)	<0.0001	12.2 ± 2.8	<0.0001
Others (obstructive, metabolic, and others)	65 (22.5%)	19 (29.2%)	<0.0001	19.3 ± 3.94	<0.0001
Undetermined	29 (10%)	10 (34.4%)	<0.0001	29.9 ± 9.15	<0.0001

*Number of patients; **mean ± standard error.

**Table 2:** Distribution on age range of transplanted patients.

Age range (years)	*N*	Positive	Odds ratio	*P* value
20–30	21	4 (19.0%)	17.02	<0.0001
30–40	42	8 (19.0%)	17.02	<0.0001
40–50	58	14 (21.1%)	23.02	<0.0001
50–60	55	18 (32.7%)	35.19	<0.0001
Over 60	112	37 (33.0%)	35.68	<0.0001

**Table 3:** Characteristics of patients with or without persistent antibodies IgA aB2GPI after transplantation.

	Patients who negativice antibodies after transplantation (*N* = 53)		Patients with persistent antibodies IgA aB2GPI (*N* = 28)		*P* value
	*N*/Mean	(%)/SE	*N*/Mean	(%)/SE	
Levels of IgA aB2GPI (U/mL)					
Pretransplant	44,7	±7	91,4	±16,5	0,0133
Posttransplant	7,1	±0,6	69,6	±13,2	<0,0001
Difference pre-/posttransplant	−37,6	±7	−21,9	±12,7	0,2403
Pretransplant situation					
Waiting time (months)	16,7	±2,4	21,9	±3,7	0,5570
Age (years)	53,5	±2,1	63,8	±2,3	0,0034
Previous transplants	4	(6,3%)	2	(7,1%)	0,7514
Induction treatment	42	(66,6%)	19	(67,8%)	0,8965
Timoglobuline	26	(41,2%)	8	(28,5%)	0,3571
Baxiliximab	16	(25,3%)	11	(39,2%)	0,2757
Etiology of endstage renal disease					
Diabetic nephropathy	14	(22.2%)	10	(35.7%)	0.2756
Glomerular disease	6	(9.5%)	2	(7.1%)	0.9754
Nephroangiosclerosis	2	(3.2%)	1	(3.6%)	1,0000
Systemic Lupus Erythematosus	1	(1.6%)	0	(0.0%)	1,0000
Interstitial nephritis	4	(6.3%)	6	(21.4%)	0.0785
Polycystic kidney disease	8	(12.7%)	2	(7.1%)	0.6752
Others	5	(7.9%)	1	(3.6%)	0.7514
Undetermined	13	(20.6%)	6	(21.4%)	0.8466
